# Supplementary material for: Experiences of adults who survived suicide attempts in rural Uganda: Stigma, support systems and reintegration
Source: Glob Ment Health (Camb). 2026 Feb 27;13:e55. doi: 10.1017/gmh.2026.10171 (PMC13112284; doi:10.1017/gmh.2026.10171)
Supplement: Lee et al. supplementary material [file S205442512610171Xsup001.docx]

**People with Suicide Attempt**

**In-Depth Questionnaire**

1. Tell us about what happened to cause your suicide attempt? (be sure to give emotional comfort as needed)
2. Who did you tell that you were thinking of killing yourself? What were their reactions?
3. What happened after you attempted suicide?
4. What was the reaction of people around you after they discovered your suicide attempt?
5. What was your family’s reaction when discovering your suicide attempt? If they did not know, what do you imagine the reaction would have been?
6. How has your suicide attempt changed your relationship with your family?
7. What was your community’s reaction when discovering your suicide attempt? If they did not know, what do you imagine the reaction would have been?
8. How has your suicide attempt changed your relationship with your community?
   1. What was the immediate community reaction after the attempt? What was the community reaction a few weeks / months later?
9. Do you feel as connected to your community compared to before?
10. How do people in the community view you after they discovered that you have attempted suicide?
11. How could people around you support you more?
12. What legal consequences, if any, were there to your suicide attempt?
13. Have the social and/or legal consequences from your suicide attempt made you want to kill yourself?
14. What are your thoughts on suicide being illegal ?
15. What has been helpful in stopping yourself from attempting suicide again?
16. If it comes up:
    1. Ex. Can the spirit of someone who committed suicide inhabit someone else
17. If the patient has mental illness - do you think the mental illness contributed to the suicide attempt?

**Families of People who Attempted Suicide**

**In-Depth Questionnaire**

1. Tell us about what happened to cause your family member’s suicide attempt
2. What happened after your family member attempted suicide?
3. What was your reaction when discovering your family member’s suicide attempt?
4. What is the role of the family in helping someone who attempted suicide?
5. How has your family member’s suicide attempt changed your relationship with them?
6. How did your community treat your family member when they found out about their suicide attempt? If they did not find out, how do you think they would have reacted if they had found out?
7. How did your community treat your family when they found about about your family member’s suicide attempt? If they did not find out, how do you think they would have reacted if they had found out?
   1. What was the immediate community reaction after the attempt? What was the community reaction a few weeks / months later?
8. What legal consequences, if any, were there to your family member’s suicide attempt?
   1. What are your thoughts on suicide being illegal ?
9. What can help someone who has thoughts of attempting suicide?
10. What supports do someone that attempted suicide need?
11. If it comes up:
    1. Ex. Can the spirit of someone who committed suicide inhabit someone else
12. If the patient has mental illness - do you think the mental illness contributed to the suicide attempt?
13. If counseling is mentioned - Can you explain what you mean by counseling? What is the meaning of that to you?

**Healthcare Worker Suicide Attempt Exposure**

**In-Depth Questionnaire**

1. Tell us about a time that you cared for someone after their suicide attempt?
2. What were some of the reasons that they attempted suicide?
3. What were your reactions to what they attempted? Do you think you treated them like any other patient?
4. What were the family’s reactions?
5. How should people that attempted suicide be treated by their family?
6. What were other healthcare workers’ reactions? Do you think they treated them like any other patient?
7. What were the community’s reactions?
8. How should people that attempted suicide be treated by their community?
9. Did the patient return to their previous living situation? If not, how did it change?
10. What happened to the patient after their hospitalization?
11. What were your future interactions with the patient, if any?
12. Do you think the rates of suicide are similar or different between men and women? If they are different, why do you think so?

**VHT Suicide Attempt Exposure**

**In-Depth Questionnaire**

1. Tell us about your experience encountering or helping a community member who attempted suicide.
2. What was your reaction in finding out about your community member attempting suicide?
3. Do you think you treated them like any other community member?
4. How did the community react to the suicide attempt survivor?
5. What next steps did you take upon hearing about your community member attempting suicide?
6. Can you describe how the suicide attempt survivor has progressed since their attempt (personal interactions you’ve had, village gossip)?
7. Do you feel it is your responsibility to help a community member who has attempted suicide?
8. What could be the reasons that someone attempts suicide?
9. Between men and women, who do you feel experiences higher rates of attempting suicide? If men, why? If women, why?
10. What do you think should be done to prevent people from attempting suicide?
